# Device-evaluated autonomic nervous function for predicting ventricular arrhythmias and all-cause mortality in patients who underwent cardiac resynchronization therapy-defibrillator

**DOI:** 10.3389/fphys.2023.1090038

**Published:** 2023-02-02

**Authors:** Chendi Cheng, Jiang Jiang, Keping Chen, Wei Hua, Yangang Su, Wei Xu, Xiaohan Fan, Yan Dai, Shu Zhang

**Affiliations:** ^1^ State Key Laboratory of Cardiovascular Disease, Arrhythmia Center, National Center for Cardiovascular Diseases, Fuwai Hospital, Chinese Academy of Medical Sciences and Peking Union Medical College, Beijing, China; ^2^ Cardiology, Shanghai Institute of Cardiovascular Diseases, Zhongshan Hospital, Fudan University, Shanghai, China; ^3^ Department of Cardiology, Nanjing Drum Tower Hospital, Nanjing, China

**Keywords:** Heart rate variability (HRV), autonomic nervous system (ANS), cardiac resychronization therapy-defibrillator, ventricular tachiarrhythmias, remote home monitoring (RHM), heart rate (HR)

## Abstract

**Introduction:** Autonomic nervous system (ANS) function quantified by heart rate variability (HRV) was associated with long-term prognosis, but it was rarely used in the evaluation of patients with heart failure, especially those with cardiac resynchronization therapy-defibrillator (CRT-D) implantation. This study aimed to describe the changes in ANS function among patients who underwent CRT-D with remote home monitoring function, and explore predictive value of HRV for ventricular tachyarrhythmias (VTAs) and all-cause mortality.

**Method:** Patients who underwent CRT-D implantation were included. Device-measured all-day HR, night-time HR, and HRV (measured by the standard deviation of the atrial–atrial sensed intervals) were used to quantify ANS function. Multivariate Cox proportional hazards models were fitted to calculate hazard ratios (HRs) and 95% confidence intervals (CIs) of VTAs or all-cause mortality in relation to ANS function at baseline and 6 months post-implantation. The cutoff value was determined using restrictive cubic splines. Multivariable logistic regression was further established to determine factors influencing postoperative HRV.

**Results:** A total of 170 patients treated with CRT-D were eligible for analysis. During a median follow-up period of 50.8 months, 61 patients died and 69 patients experienced at least one spontaneous episode of VTAs. At 6 months after CRT implantation, 114 patients showed improvement in HRV, increasing from 66.4 ± 19.4 ms to 76.7 ± 21.2 ms. The postoperative HRV was associated with both all-cause mortality (HRs: 0.983; 95% CI: 0.968 to 0.998, *p* = 0.012) and VTAs (HRs: 0.973; 95% CI: 0.954 to 0.993, *p* = 0.008), and the relative risk would significantly increase when the postoperative HRV lower than 75 ms. After adjusting for basic ANS function and possible influencing factors, patients without diabetes (*p* = 0.018) and with higher daily physical activity (*p* = 0.041) could maintain higher postoperative HRV after CRT implantation.

**Conclusion:** More than two-thirds of heart failure patients showed improvement in ANS function following CRT treatment. However, patients with diabetes and low daily physical activity levels have difficulty maintaining a higher postoperative HRV, which is associated with a worse clinical outcome.

## 1 Introduction

The heart is innervated by the autonomic nervous system (ANS) including the sympathetic and the parasympathetic nervous system. ANS dysfunction plays a central role in the pathophysiology of myocardial remodeling, deterioration of left ventricular function, and ventricular tachyarrhythmias (VTAs) (Goldberger et al., 2019; Stavrakis et al., 2020). Also, a variety of cardiac pathologies, such as myocardial infarction, heart failure (HF), and cardiomyopathy may promote both anatomic and functional changes in the cardiac ANS. These changes may, in turn, contribute to the progression of the disease or be involved in arrhythmogenesis (Goldberger et al., 2019; Stavrakis et al., 2020).

Clinically, changes in heart rate (HR) are generally regarded as a direct reflection of sympathetic or parasympathetic nerve excitation. In particular, the mean HR evaluated under resting and steady state may better reflect the cardiac autonomic nervous function (Lahiri et al., 2008; Goldberger et al., 2019). Based on the changes in HR, several non-invasive techniques are available to assess autonomic function such as heart rate variability (HRV), heart rate turbulence, and baroreflex sensitivity (Lahiri et al., 2008; Goldberger et al., 2019; Stavrakis et al., 2020). HRV represents a measure of the oscillation in the intervals between consecutive heart beats and reflects the balance between sympathetic and parasympathetic mediators. High HRV has been associated with better cardiac status (Jensen-Urstad et al., 1997), whereas low HRV has been correlated with an increased risk of lethal cardiac arrhythmias and sudden death (La Rovere et al., 2003; Deyell et al., 2015). The traditional method of assessing HRV depends on surface 24-h Holter monitoring, without the possibility of longer-term recordings. An implantable device can continuously monitor HR and provide more accurate HRV data. In previous clinical studies, HRV has been used to quantify the ANS function for HF patients who underwent cardiac resynchronization therapy (CRT) (Fantoni et al., 2005; Gilliam et al., 2007; Landolina et al., 2008), and CRT is demonstrated that can significantly modify the sympathetic-parasympathetic interaction with the heart, as evidenced by elevated HRV (Fantoni et al., 2005; Gilliam et al., 2007). However, the long-term clinical benefit brought from improved HRV has rarely been evaluated and discussed in HF patients after CRT-defibrillator (CRT-D) implantation. It has shown that patients with severe autonomic nervous dysfunction have only limited benefits from CRT (Sherazi et al., 2015), but other possible factors affecting the recovery of ANS function have not been well identified. This study aims to explore the changes in ANS function among HF patients after CRT-D implantation with a home-monitoring system, as well as the possible influencing factors. To explore its predictive value for VTAs and all-cause mortality.

## 2 Materials and methods

### 2.1 Study population

The Study of Home Monitoring System Safety and Efficacy in Cardiac Implantable Electronic Device-implanted Patients (SUMMIT) registry was an observational, prospective, and multicenter trial. We retrospectively analyzed archived home-monitored transmission data from the SUMMIT registry. The present study complied with the Declaration of Helsinki and was approved by the ethics committee of Fuwai Hospital and all other participating organizations. All patients provided written informed consent before entering the study.

### 2.2 Patient selection

Patients who underwent CRT-D implantation (Biotronik, Berlin, Germany) were included upon meeting the following inclusion criteria: 1) Primary prevention for sudden cardiac death; 2) The remote home-monitoring system was continuously working and data were available during the target window. The exclusion criteria were as follows: 1) Age at CRT-D device implantation was <18 years; 2) during the target monitoring window, the average atrial fibrillation burden or atrial pacing percentage >10%; and 3) the postoperative survival period was less than 6 months, or the patient received heart transplantation.

### 2.3 Autonomic nervous function assessment

Three device-measured parameters obtained from the remote home-monitoring system were used to quantify autonomic nervous function, including all-day HR (AHR), night-time HR (NHR), and daily HRV. Specifically, AHR was obtained from a 24-h period, while NHR was obtained from 2:00 a.m. to 6:00 a.m. when patients were at a sleep status. HRV was measured by a time domain analysis and quantified as the standard deviation of the atrial–atrial sensed intervals (SDANN) over a 24-h period. Paced atrial beats, supraventricular and ventricular premature beats, and arrhythmic episodes were automatically excluded from the analysis by the device. The technical feasibility of the SDANN measurement achieved by automatic algorithms of devices has been previously demonstrated (Malik et al., 1999). The average data (AHR, NHR, and HRV) from the first month (1–30 days) post-implantation was collected as the baseline. To investigate the changes in ANS function, data from the 6th month (150–180 days) following CRT implantation were collected, as this period commonly determines the effect of CRT (Ellenbogen and Huizar, 2012).

### 2.4 Data collection and follow-up

A total of 170 patients who received CRT-D with a remote home monitoring system were eligible for analysis. Data about demographic variables (e.g., sex and age at device implantation), clinical complications (e.g., etiology, hypertension, and diabetes), echocardiographic parameters, history of percutaneous coronary intervention (PCI) or coronary artery bypass grafting (CABG), and medication intake were collected from medical history before discharge. Daily physical activity (PA) was measured by an accelerometer sensor embedded in the device every day, and any acceleration above 0.473 m/s^2^ was recognized as an activity. Previous studies have also proved that acceleration sensors were highly sensitive in detecting PA (Conraads et al., 2014). During the follow-up, the parameters mentioned for the assessment of autonomic nervous function and daily PA were automatically collected and transmitted daily to the service center through a remote home-monitoring system. If data transmission was interrupted for more than 24 h, clinical specialists would contact the patient and family members to confirm the monitoring function of the device, as well as the condition of the patient. Routine telephone follow-up was also conducted to collect clinical information regarding the clinical outcomes. All VTAs events (Ventricular tachycardia and fibrillation) detected by devices were re-confirmed by physicians. The date of death was confirmed by medical records or death certificates.

### 2.5 Statistical analysis

Categorical variables, presented as numbers with relative percentages, were compared using the chi-squared test. Continuous variables are expressed as mean ± standard deviation or median (interquartile range [IQR]). Comparisons between different groups were performed using Student’s *t*-test. A paired *t*-test was used to compare differences in ANS parameters between different periods. Box plots and Histograms were used to describe the variation of autonomic nervous function parameters between baseline and 6 months follow-up. Univariable and Multivariate Cox proportional hazards models were fitted to calculate hazard ratios (HRs) and 95% confidence intervals (CIs) of VTAs or all-cause mortality in relation to autonomic nervous function parameters (AHR, NHR, and HRV) at baseline and 6 months post-implantation. Significant univariate and traditional risk factors were adjusted (e.g., age at implantation, gender, body mass index [BMI], daily PA, left ventricular ejection fraction [LVEF], etiology, history of PCI or CABG, hypertension, and diabetes). The cutoff value was further determined using restrictive cubic splines. Based on the cutoff value of HRV, patients were divided into two groups: high-level HRV and low-level HRV groups. Kaplan–Meier curves were constructed to compare event rates between different groups and were formally assessed using log-rank testing. Multivariable logistic regression, adjusting for possible confounding factors, was further established to determine factors associated with postoperative HRV.

Statistical significance was set at *p* < 0.05, and all tests were two-sided. All statistical analyses were performed using GraphPad Prism version 8.0 (GraphPad Software, San Diego, CA, United States), SPSS statistical software version 23 (IBM Corp, Armonk, NY, United States), and R version 4.0.3 (R Foundation for Statistical Computing, Vienna, Austria).

## 3 Results

### 3.1 Baseline characteristics and clinical outcome

A total of 170 patients who underwent CRT-D implantation were eligible for analysis. Table 1 summarizes the baseline characteristics of the whole population. The mean age of the participants at device implantation was 62.1 ± 12.5 years, 78.8% were men, 35.9% had hypertension, and 17.1% suffered from diabetes. The vast majority of patients (54.7%) had ischemic cardiomyopathy presenting with severely reduced LVEF, and dilated ventricles, in advanced New York Heart Association functional class despite best optimized medical therapy. Thirty-three patients (19.4%) had a history of PCI or CABG. The mean AHR and NHR at baseline were 75.3 ± 9.4 bpm and 65.1 ± 8.2 bpm, respectively. The baseline HRV was 66.7 ± 19.4 ms with daily PA of 151.1 ± 74.7 min.

**TABLE 1:** Baseline characteristics.

Characteristic	Total (n = 170)	VTAs (*n* = 69)	All-cause mortality (*n* = 61)
Clinical virables			
Age at implantation (year)	62.1 ± 12.5	60.6 ± 12.0	61.5 ± 13.7
Male (%)	134 (78.8)	57 (82.6)	47 (77.0)
BMI (kg/m^2^)	23.5 ± 2.9	23.8 ± 2.9	23.3 ± 2.4
PA (min/day)	151.1 ± 74.7	153.1 ± 75.9	124.5 ± 64.3
LVEF (%)	30.2 ± 5.9	30.0 ± 6.5	30.3 ± 5.9
LVEDD (mm)	68.2 ± 11.5	69.8 ± 12.7	67.8 ± 12.6
NYHA class III-IV (%)	142 (83.5)	56 (81.2)	55 (90.2)
Baseline ANS function			
AHR (bpm)	75.2 ± 9.4	76.3 ± 9.7	75.6 ± 10.5
NHR (bpm)	66.2 ± 8.2	67.1 ± 8.4	67.6 ± 9.1
HRV (ms)	66.4 ± 19.4	64.9 ± 18.7	60.5 ± 15.7
Comorbidities			
Ischemic cardiomyopathy (%)	93 (54.7)	40 (58.0)	34 (55.7)
PCI or CABG (%)	33 (19.4)	12 (17.4)	16 (26.2)
Hypertension (%)	61 (35.9)	23 (33.3)	29 (47.5)
Diabetes (%)	29 (17.1)	17 (24.6)	11 (18.0)
Stroke (%)	6 (3.5)	1 (1.5)	3 (4.9)
Medications			
*β* blocker (%)	157 (92.4)	64 (92.8)	57 (93.4)
ACEI/ARB (%)	145 (85.3)	57 (82.6)	50 (82.0)
Diuretics (%)	93 (54.7)	36 (52.2)	35 (57.4)
Digoxin (%)	65 (38.2)	24 (34.8)	24 (39.3)
Amiodarone (%)	34 (20.0)	12 (17.4)	15 (24.6)

AHR, all-day heart rate; ANS, autonomic nervous system; BMI, body mass index; CABG, coronary artery bypass grafting; HRV, heart rate variability; LVEDD, left ventricular end-diastolic dimension; LVEF, left ventricular ejection fraction; NHR: night-time heart rate; NYHA, New York Heart Association; PA, physical activity; PCI, percutaneous coronary intervention.

During a median follow-up period of 50.8 (IQR: 27.1–70.2) months, sixty-one patients (35.9%) died. There are 37.1% (*n* = 69) patients who experienced at least one spontaneous episode of VTAs appropriately detected and terminated by the device. The median time from implantation to the first VTAs was 32.7 (IQR: 13.8–64.2) months.

### 3.2 Changes in automatic nervous function after CRT-D implantation

**FIGURE 1:**
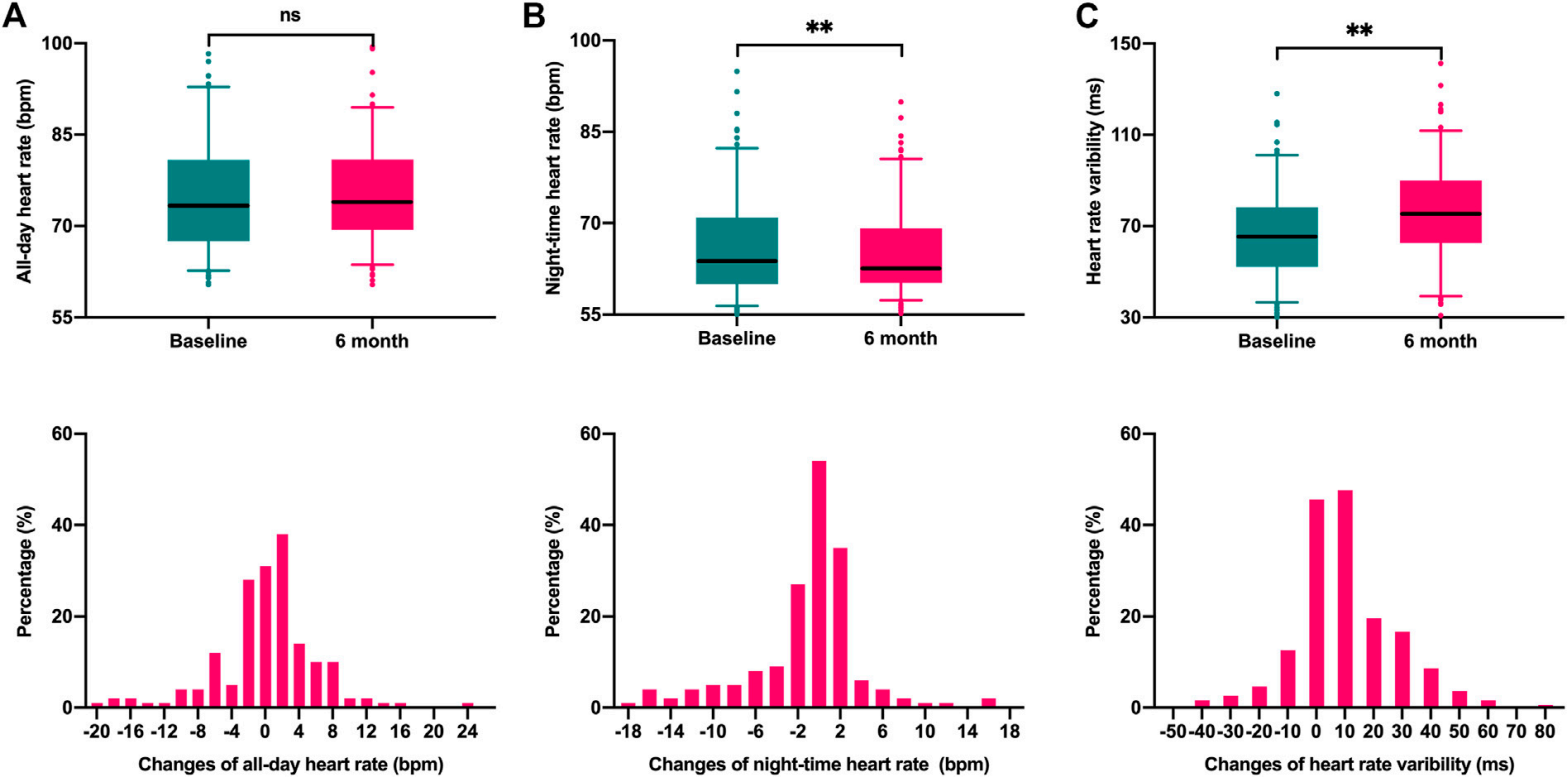
Box plots and Histograms were used to describe the variation of autonomic nervous function parameters between baseline and 6 months follow-up. **(A)**. All-day heart rate (*p* = 0.78); **(B)** night-time heart rate (*p* = 0.005); **(C)** Heart rate variability (*p* < 0.001).

Figure 1 illustrates the changes in ANS function parameters (AHA, NHR, and HRV) between baseline and 6 months after CRT implantation. HRV increased from 66.4 ± 19.4 ms at baseline to 76.7 ± 21.2 ms at 6 months after implantation (*p* < 0.001). Of the 170 patients included, the majority of patients (67.1%) had elevated HRV, ranging from 0.3 ms to 77.3 ms, while the remaining 56 (32.9%) patients showed a decrease (ranging from −0.4 ms to −42.8 ms). No significant changes in AHR relative to baseline (75.2 ± 9.4 bpm vs. 75.3 ± 8.1 bpm, *p* = 0.78), while the NHR decreased from 66.2 ± 8.1 bpm at baseline to 65.1 ± 7.0 bpm at 6 months (*p* = 0.005).

### 3.3 Predictive value of the postoperative automatic nervous function

Univariable and multivariable Cox proportional hazards models were established to assess the predictive role of automatic nervous function parameters both for VTAs and all-cause mortality. As Table 2 shown, univariable Cox analysis revealed that both baseline AHR (HRs:1.03; 95% CI: 1.005 to 1.056, *p* = 0.021) and NHR (HRs:1.037; 95% CI: 1.009 to 1.065, *p* = 0.009) were related to the VTAs. However, the correlation was no longer significant in the multivariable analysis adjusted for possible confounding factors (age at implantation, gender, BMI, NYHA functional class, LVEF, daily PA, etiology, history of PCI or CABG, hypertension, and diabetes). The baseline HRV was associated with the risk of all-cause mortality on univariable Cox analysis (HRs:0.979; 95% CI: 0.966 to 0.993, *p* = 0.003), and the relationship remains significant after adjustment for confounding factors (HRs: 0.980; 95% CI: 0.963 to 0.996, *p* = 0.018).

**TABLE 2:** Predictive roles of baseline automatic nervous function for VTAs and all-cause mortality.

	Univariate		Multivariate	
	HRs 95% CI	*p*-value	HRs 95% CI	*p*-value
VTAs				
AHR (1 bpm)	1.030 (1.005–1.056)	0.021	1.018 (0.960–1.080)	0.558
NHR (1 bpm)	1.037 (1.009–1.065)	0.009	1.002 (0.932–1.078)	0.952
HRV (10 ms)	0.990 (0.978–1.002)	0.115	0.989 (0.973–1.005)	0.170
All-cause mortalituy				
AHR (1 bpm)	1.020 (0.993–1.047)	0.151	0.981 (0.920–1.047)	0.563
NHR (1 bpm)	1.040 (1.012–1.069)	0.005	1.028 (0.946–1.116)	0.515
HRV (10 ms)	0.979 (0.966–0.993)	0.003	0.980 (0.963–0.996)	0.018

The multivariate Cox regression model was adjusted for age at implantation, gender, body mass index, daily physical activity, left ventricular ejection fraction, etiology, history of PCI, or CABG, hypertension, and diabetes; AHR, all-day heart rate; CABG, coronary artery bypass grafting; CIs: confidence intervals; HRs: hazard ratios; HRV, heart rate variability; NHR, night-time heart rate; PCI: percutaneous coronary intervention; VTAs, ventricular tachyarrhythmias.

Table 3 further lists the predictive role of postoperative automatic nervous function at 6 months following CRT implantation after adjustment for additional baseline ANS function. The postoperative HRV at 6 months was an independent predictor both for VTAs (HRs: 0.973; 95% CI: 0.954 to 0.993, *p* = 0.008) and all-cause mortality (HRs: 0.983; 95% CI: 0.968 to 0.998, *p* = 0.012). For every 10 ms increase in HRV at 6 months after CRT implantation, the risk of VTAs and death decreased by 27% and 17%, respectively. Additionally, NHR at 6 months after implantation was significantly associated with long-term survival (HRs: 1.107; 95 CI: 1.015 to 1.208, *p* = 0.033), indicating that each 1 bpm increase in NHR is linked to an additional 17% increase in the risk of all-cause mortality.

**TABLE 3:** Predictive roles of postoperative automatic nervous function for VTAs and all-cause mortality.

	Univariate		Multivariate	
	HRs 95% CI	*p*-value	HRs 95% CI	*p*-value
VTAs				
AHR (1 bpm)	1.033 (1.000–1.067)	0.051	1.045 (0.978–1.117)	0.191
NHR (1 bpm)	1.062 (1.027–1.099)	0.001	0.975 (0.896–1.061)	0.557
HRV (10 m)	0.978 (0.967–0.990)	0.001	0.973 (0.954–0.993)	0.008
All-cause mortalituy				
AHR (1 bpm)	1.028 (0.996–1.062)	0.087	0.959 (0.897–1.026)	0.223
NHR (1 bpm)	1.068 (1.035–1.103)	0.001	1.107 (1.015–1.208)	0.033
HRV (10 ms)	0.973 (0.961–0.985)	0.001	0.983 (0.968–0.998)	0.012

The multivariate Cox regression model was adjusted for age at implantation, gender, body mass index, daily physical activity, left ventricular ejection fraction, etiology, baseline automatic nervous function, history of PCI, or CABG, hypertension, and diabetes; AHR, all-day heart rate; CABG, coronary artery bypass grafting; CIs: confidence intervals; HRs: hazard ratios; HRV, heart rate variability; NHR, night-time heart rate; PCI: percutaneous coronary intervention; VTAs, ventricular tachyarrhythmias.

**FIGURE 2:**
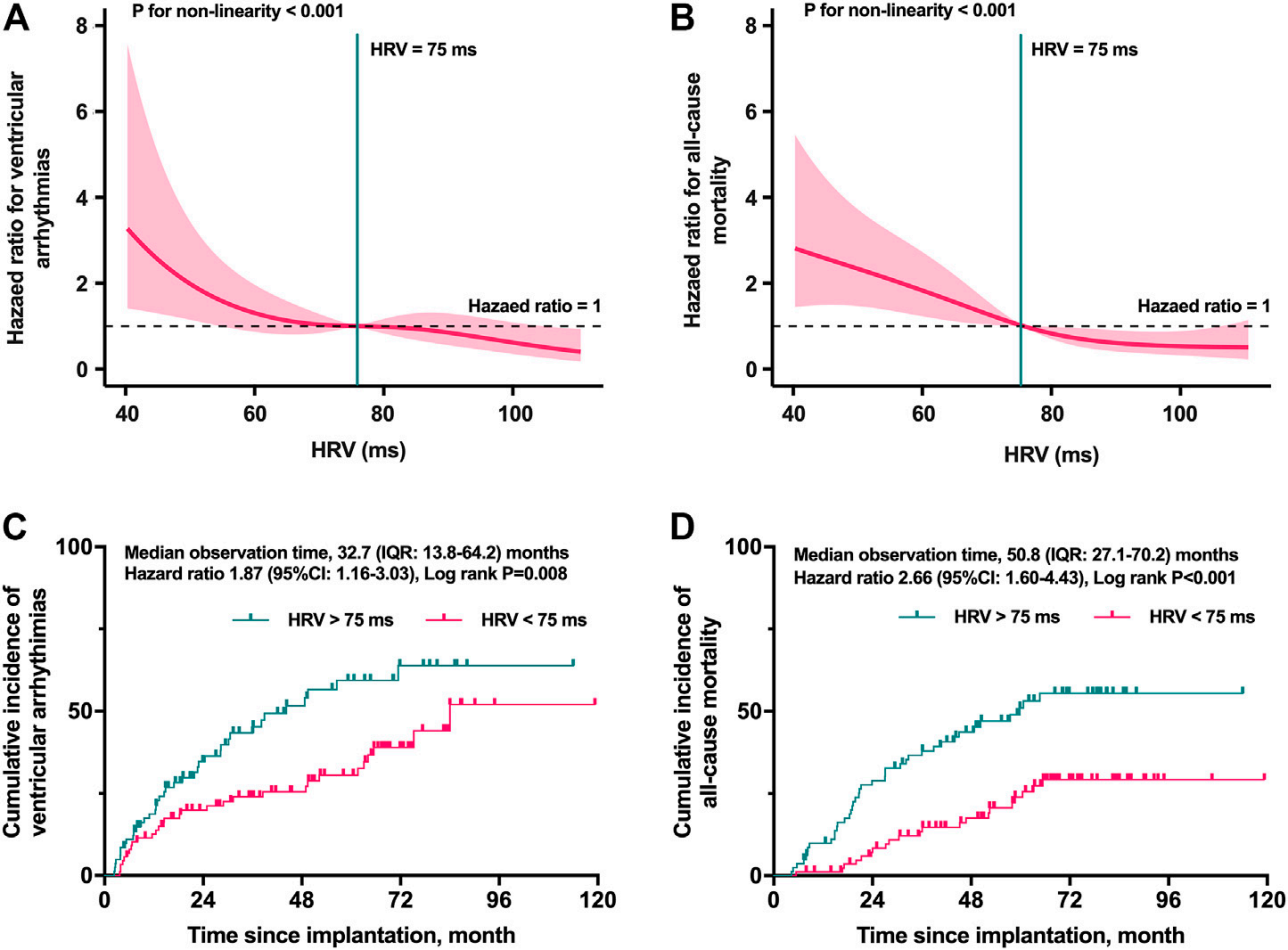
Restricted cubic spline plots for selecting the cut-off value and the Kaplan–Meier curves stratified by the cut-off value. The risk of both VTAs **(A)** and all-cause mortality **(B)** were relatively flat when the postoperative HRV fluctuated around 75 ms (p for non-linearity <0.001). The cumulative incidence of VTAs **(C)** and all-cause mortality **(D)** in different levels of postoperative HRV. HRV, heart rate variability; IQR, interquartile range; VTAs, ventricular tachyarrhythmias.

The smooth curve fitting was utilized for assessing the associations between the postoperative HRV and HRs for VTAs and all-cause mortality. As shown in Figures 2A, B, the risk of both VTAs and all-cause mortality were relatively flat when the postoperative HRV fluctuated around 75 ms. However, the risk elevated rapidly when the postoperative HRV was lower than 75 ms (p for non-linearity <0.001). Depending on the level of postoperative HRV, the cumulative incidence of VTAs (HRs: 1.68; 95% CI: 1.05 to 2.71, log-rank *p* = 0.028) and all-cause mortality (HRs: 2.42; 95% CI: 1.46 to 4.01, log-rank *p* < 0.001) were shown in Figures 2C, D, respectively.

### 3.4 Factors affecting the postoperative automatic nervous function

**FIGURE 3:**
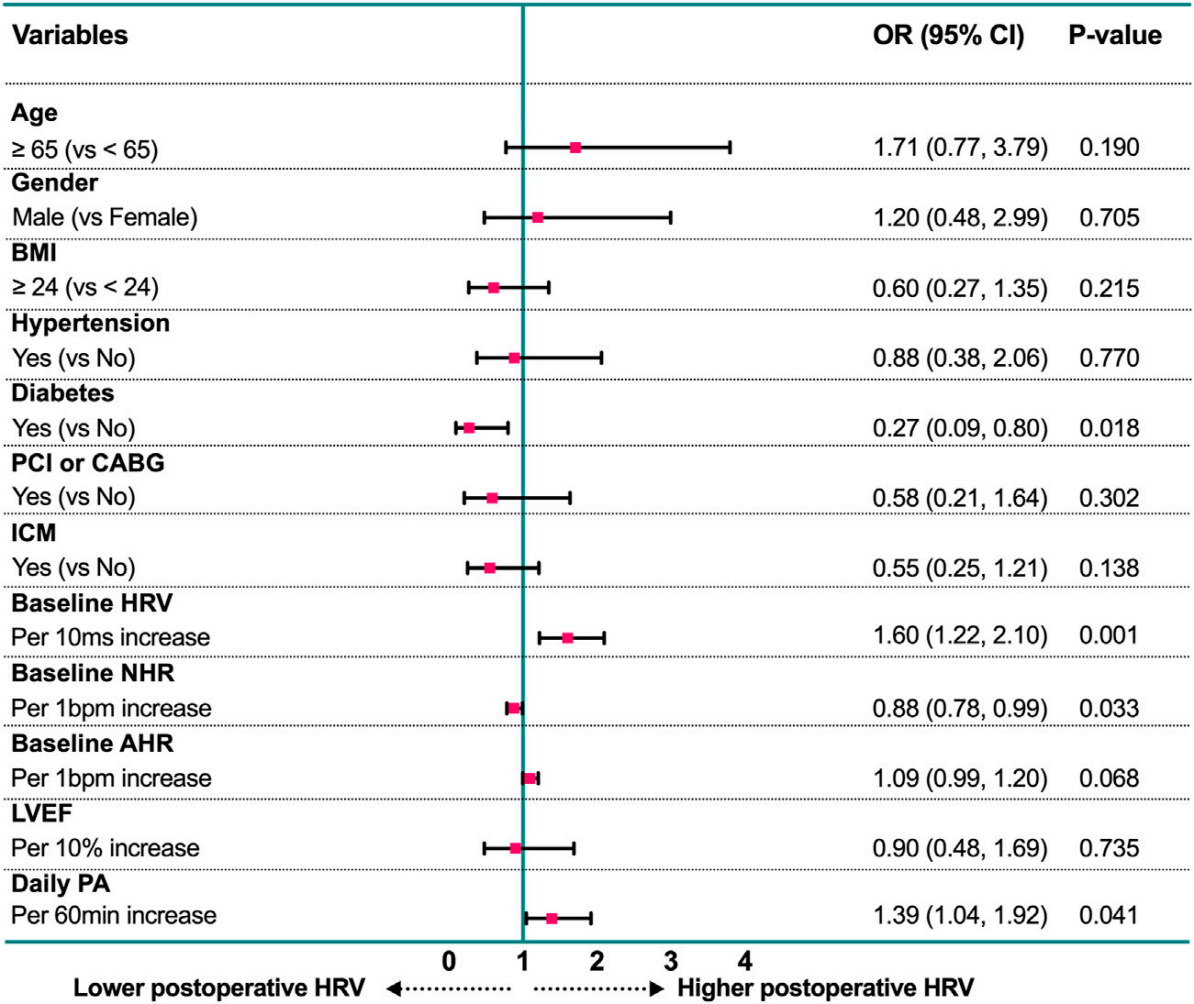
Forest plot of estimates from multivariable logistic regression analysis for prediction of postoperative HRV according to potential factors. AHR, all-day heart rate; BMI, body mass index; CABG, coronary artery bypass grafting; HRV, heart rate variability; ICM: Ischemic cardiomyopathy; LVEF, left ventricular ejection fraction; NHR: night-time heart rate; PA, physical activity; PCI, percutaneous coronary intervention.

Based on the cutoff value of postoperative HRV at 6 months after CRT implantation, patients were divided into two groups: The high-level group (HRV ≥ 75 ms, *n* = 88) and the low-level group (HRV < 75 ms, *n* = 82). The baseline characteristics of the two groups were summarized in Table 4. In addition to the differences in the basic autonomic function (HRV and NHR, both *p* < 0.001), patients who maintain a higher level of postoperative HRV have higher daily PA (*p* = 0.007) activity and lower BMI (*p* = 0.008), and are less likely to be complicated with ischemic cardiomyopathy (*p* = 0.028) and diabetes (*p* = 0.014). As shown in Figure 3, in the multivariable logistic regression model (adjusted for age, gender, BMI, daily PA, baseline ANS function, LVEF, and comorbidities), diabetes is still an independent risk factor for the lower postoperative HRV after CRT (OR: 0.27, 95% CI: 0.09 to 0.80, *p* = 0.018). Patients with a high level of daily PA could maintain a higher postoperative HRV (OR: 1.39, 95% CI: 1.04 to 1.92, *p* = 0.041).

**TABLE 4:** Baseline characteristics for patients with different level of postoperative HRV.

Characteristic	High level group (≥75 ms, *n* = 88)	Low level group (<75 ms, *n* = 82)	*p*-value
Clinical virables			
Age (year)	61.8 ± 13.0	62.5 ± 11.9	0.722
Male (%)	68 (77.3)	66 (80.5)	0.608
BMI (kg/m^2^)	22.9 ± 2.6	24.1 ± 3.1	0.008
PA (min/day)	165.9 ± 82.5	135.1 ± 61.8	0.007
LVEF (%)	30.2 ± 5.9	30.1 ± 5.8	0.896
LVEDD (mm)	67.9 ± 10.7	68.6 ± 12.4	0.723
NYHA class III-IV(%)	72 (81.8)	70 (85.4)	0.533
Baseline ANS function			
AHR (bpm)	74.4 ± 8.5	76.0 ± 10.2	0.286
NHR (bpm)	64.1 ± 6.9	68.6 ± 8.9	<0.001
HRV (ms)	56.9 ± 14.8	75.2 ± 19.1	<0.001
Comorbidities			
Ischemic cardiomyopathy (%)	41 (46.6)	52 (63.4)	0.028
PCI or CABG (%)	15 (17.0)	18 (22.0)	0.419
Hypertension (%)	27 (30.7)	34 (41.5)	0.143
Diabetes (%)	9 (10.2)	20 (24.4)	0.014
Stroke (%)	5 (3.5)	1 (0)	0.246
Medications			
*β* blocker (%)	82 (93.2)	75 (91.5)	0.674
ACEI/ARB (%)	75 (85.2)	70 (85.4)	0.980
Diuretics (%)	46 (52.3)	47 (57.3)	0.509
Digoxin (%)	36 (40.9)	29 (35.4)	0.457
Amiodarone (%)	16 (18.2)	18 (22.0)	0.539

AHR, all-day heart rate; ANS, autonomic nervous system; BMI, body mass index; CABG, coronary artery bypass grafting; HRV, heart rate variability; LVEDD, left ventricular end-diastolic dimension; LVEF, left ventricular ejection fraction; NHR: night-time heart rate; NYHA, New York Heart Association; PA, physical activity; PCI, percutaneous coronary intervention.

## 4 Discussion

In this study, the remote home monitoring system was used to dynamically evaluate the ANS function in HF patients who underwent CRT implantation by continuously monitoring AHR, NHR, and HRV. The main findings were as follows: 1) At 6 months after CRT-D implantation, more than two-thirds of the patients showed improvement in HRV. 2) Postoperative HRV was significantly associated with both all-cause mortality and VTAs. Moreover, the relative risk would be obviously increased in those with a lower level of HRV (<75 ms) after 6 months of CRT-D implantation. 3) After adjusting the baseline HRV and clinical characteristics, patients without diabetes and with higher daily PA could maintain higher postoperative HRV.

In clinical practice, cardiac autonomic control can be usually quantified by HRV and mean HR, commonly measured by 24-h Holter (Lahiri et al., 2008; Goldberger et al., 2019; Stavrakis et al., 2020). However, the result of a single-point measurement may be biased, and it may have limited applicability for repeated measurements in daily practice. In contrast, the parameters detected by cardiac implantable electronic devices with remote home monitoring systems can dynamically reflect the changes in ANS function. Several studies have revealed the relationship between HRV parameters and increases in VTAs among patients with implantable cardioverter defibrillators (Battipaglia et al., 2010; Battipaglia et al., 2012). For HF patients with ANS dysfunction, the diminished HRV can be monitored by CRT (Braunschweig et al., 2005; Fantoni et al., 2005; Gilliam et al., 2007; Landolina et al., 2008). In the present study, HRV was analyzed as the time-domain parameter SDANN with a mean value of 66.4 ± 19.4 ms, which was similar to 69.2 ± 25.5 ms reported by Gilliam et al. (2007) and 64 ± 23 ms reported by Braunschweig et al. (2005). Also, CRT was demonstrated that can provide a favorable impact on cardiac autonomic control, evidenced by the improved HRV parameters after CRT (Adamson et al., 2004; Fantoni et al., 2005; Gilliam et al., 2007; Landolina et al., 2008). We also found that there are more than two-thirds of the patients showed improvement in HRV 6 months after CRT-D implantation. The mean NHR also decreased. These may benefit from the improved hemodynamics and reversal of ventricular remodeling and may contribute to improvement in long-term prognosis.

Although an increase in HRV or a decrease in NHR indicates the improvement of ANS function, its clinical benefit has rarely been evaluated in patients treated with CRT-D. Previous studies have described the predictive role of the baseline (Sherazi et al., 2015) or short-term changes of HRV (La Rovere et al., 2003; Fantoni et al., 2005) in patients with HF treated with CRT. However, as shown above, there are more than two-thirds of the patients showed significant improvement in HRV at 6 months. Compare with the baseline HRV, the postoperative HRV after CRT-D may be more meaningful. Thus, we further assessed the predictive value of postoperative HRV at 6 months for long-term survival and risk of VTAs. For every 10 ms increase in HRV at 6 months after CRT-D implantation, the risk of all-cause mortality and VTAs decreased by 27% and 17%, respectively. Moreover, For those with a lower level of postoperative HRV (<75 ms), both the risk of all-cause mortality and VTAs increased significantly. It also supports that patients with little improvement in ANS function after CRT may contribute to a poor prognosis.

Additionally, few studies (Gilliam et al., 2007) have assessed the clinical characteristics that may affect the improvement of HRV after CRT-D implantation. In the present study, after the adjustment for basic ANS function, we found that HF patients with diabetes have still very limited improvement in autonomic nervous function after CRT. Usually, diabetic patients may have severely impaired cardiac ANS function and sympathovagal imbalance, especially those with a mitochondrial DNA mutation (Momiyama et al., 2002). It suggests that the damage to cardiac ANS function caused by diabetes may be irreversible, and it may also be one of the reasons why HF patients with diabetes benefit less from CRT (Sardu et al., 2017). On the contrary, patients with a higher level of daily PA can maintain higher postoperative HRV levels. The improvement of ANS function promoted by exercise may also be an indirect reason for the improvement in prognosis by regular exercise (Cheng et al., 2022). However, hypertension and ischemic heart disease were also considered to be associated with baseline ANS function in the general population (Weiss et al., 1991; Tegegne et al., 2020), but they did not play decisive roles in postoperative HRV for HF patients with CRT-D. Nevertheless, the recovery of ANS function in patients with diabetes needs to be further explored, it is clear that the ANS function of these patients deserves further attention, and improving daily activities may help to benefit more from CRT-D.

## 5 Limitation

Some possible limitations of our study need to be stated. First, all data were collected retrospectively, a larger sample study is needed for prospective validation, especially the baseline ANS function assessment is more convincing if done before device implantation. In addition, to avoid possible bias caused by single-point measurements, monthly averages were used in this study. However, this method may obscure some confounding factors, such as circadian rhythm and weekend. Caution should be exercised when interpreting and quoting conclusions. The current device algorithm still needs to be further improved, such as automatically calculating HRV in different periods to further explore the related circadian rhythm changes. Third, there is a lack of systematic evaluation of CRT efficacy at 6 months after implantation, so the specific relationship between improved ANS function and CRT response cannot be determined. Although diabetes and low-level daily PA were determined that associated with lower postoperative HRV, more details about diabetes and PA, such as glycemic control and exercise patterns, need to be revealed.

## 6 Conclusion

More than two-thirds of the heart failure patients showed improvement in ANS function following 6 months of CRT-D treatment. However, patients with diabetes and low-level exercise showed limited improvement and have difficulty maintaining a higher postoperative HRV (SNADD <75 ms), which would be accompanied by a significantly higher risk of both VT and all-cause mortality.

## Data Availability

The raw data supporting the conclusion of this article will be made available by the authors, without undue reservation.
